# The Heterogeneity of Reading and Spelling Deficits in Posterior Cortical Atrophy

**DOI:** 10.3390/brainsci15111154

**Published:** 2025-10-28

**Authors:** Nneka Watson, Megan Quimby, Daisy Hochberg, Bradford C. Dickerson, Deepti Putcha

**Affiliations:** 1 Frontotemporal Disorders Unit, Alzheimer’s Disease Research Center, Department of Psychiatry and Neurology, Massachusetts General Hospital, Boston, MA 02129, USA; nwatson@mgh.harvard.edu (N.W.); mquimby@mgb.org (M.Q.); dhochberg@mgh.harvard.edu (D.H.); brad.dickerson@mgh.harvard.edu (B.C.D.); 2 Department of Neurology and Psychiatry, Harvard Medical School, Boston, MA 02115, USA; 3 Center for Brain Mind Medicine, Brigham & Women’s Hospital, Boston, MA 02115, USA

**Keywords:** atypical AD, posterior cortical atrophy, language, neuropsychology, cognition, visuospatial, neurodegeneration, heterogeneity, reading, spelling

## Abstract

Background: Posterior Cortical Atrophy (PCA) is a clinical syndrome marked by progressive visuospatial impairment, usually due to underlying Alzheimer’s disease. While reading and spelling deficits are recognized clinical features of this syndrome, the contributions of visuoperceptual versus linguistic deficits to these impairments are still unclear. Methods: To that end, we examined reading and spelling performance in 23 individuals from the Massachusetts General Hospital PCA cohort. Participants completed tests of reading from the Western Aphasia Battery and spelling to dictation from the Boston Diagnostic Aphasia Examination. A mixed-methods analysis included quantitative scoring and qualitative observations of visual behaviors, error patterns, and compensatory strategies. Results: Participants commonly demonstrated visual scanning errors, difficulty following multi-line text, and spelling errors reflecting both visual–perceptual and orthographic–linguistic breakdowns. Conclusions: Because reading and spelling in PCA are variably impaired cognitive skills driven by visual deficits and lexical vulnerability, assessments and interventions must account for deficits in both cognitive processes. Our findings highlight the vulnerability of reading and spelling in PCA and underscore the need for multimodal assessment strategies that account for the interplay of visual, phonological, and lexical processes. These insights can inform diagnosis and guide the development of accessible interventions tailored to optimize compensatory strategies to support functional language abilities.

## 1. Introduction

Posterior Cortical Atrophy (PCA) is a clinical syndrome characterized by a progressive decline in visuospatial and visuoperceptual skills due to neurodegeneration in the occipito-parietal and occipito-temporal cortices of the brain [1,2]. This syndrome has been referred to as the “visual variant” of Alzheimer’s disease (AD) [3,4], as the vast majority (>94%) of individuals with PCA syndrome have been found to have underlying AD pathology [5]. Though the primary domain of impairment in PCA is visuospatial cognition, deficits in language have also been described as occurring relatively early in the illness trajectory [6,7,8]. The literature describes a so-called “logopenic” syndrome in PCA, highlighting deficits in lexical–semantic word retrieval in the form of verbal fluency and naming deficits [6,7,9,10], phrase length-dependent repetition [7], slowed speech [11], and phonological errors [8]. Spontaneous speech deficits have also been reported in the early symptomatic stages of PCA and are characterized by higher word frequency and fewer relational word use when describing a pictured scene [12].

Individuals with PCA syndrome also experience unique challenges with reading and orthography. Indeed, alexia and agraphia are some of the core cognitive features identified as part of current PCA consensus diagnostic criteria [1]. Severe dyslexia is common in this syndrome, thought to be present in most individuals with PCA [2,13], even occurring early in the course of illness [14]. Specifically, accounts of reading and writing abilities have been generally described in PCA by case studies highlighting impairment in pseudoword reading, word recognition, reading passages with line breaks, word omissions impacting reading comprehension, peripheral agraphia (e.g., inappropriate spacing between letters), and central agraphia (e.g., letter transpositions) [6,13,15]. There is converging evidence that visuospatial deficits undermine reading accuracy in PCA [16,17] and that interventions aimed at presenting words in smaller print and letters presented in isolation or in the context of high contrast improve reading performance [18,19]. Less is known about spelling abilities in PCA; this skill is often investigated with a focus on agraphia as difficulty with letter formation rather than pure spelling impairment [1,6]. As PCA progresses, the degradation of reading and spelling abilities significantly impact a patient’s communication and quality of life. Recent research underscores the need for targeted assessments that differentiate between the visual and linguistic components of reading impairments [20].

From a neuroanatomical perspective, the brain regions impacted at the earliest stages of PCA that underlie visuospatial cognition are also essential for the orthographic processing necessary for accurate spelling, making patients particularly susceptible to graphemic buffer impairments related to spatial agraphia [6]. Studies of the neural mechanisms underlying reading and spelling impairments have highlighted the key role of a region within the right occipito-temporal cortex (the visual word form area) and the inferior longitudinal fasciculus (ILF) as critical to linking visual inputs and object representations to their lexical labels [21,22,23]. Both the right occipito-temporal cortex and the ILF are often impacted early in the course of PCA [1,24,25,26].

The convergence of anatomical findings with model-based frameworks for reading and spelling may help clarify impairments at early visual, orthographic, and phonological levels in individuals with PCA. For example, the Dual-Route Cascaded (DRC) model describes two interacting pathways for processing written material. The lexical route enables rapid recognition and retrieval of both regular and irregular *familiar* words, whereas the non-lexical route converts graphemes to phonemes to support the reading of *unfamiliar* and regular words that follow consistent spelling to sound correspondences [27]. The model posits that distinct patterns of reading and spelling impairment can emerge depending on which route is disrupted or degraded [27,28].

The lexical route, which typically allows fast recognition for reading and spelling familiar words, may be compromised in PCA due to degradation in visual–orthographic processing which involves rapid and early recognition of written word forms. This process is subserved by specialized neural regions including the visual word form area in the left lateral occipitotemporal cortex [27,28]. This lexical route relying on visual–orthographic processing differs from grapheme-to-phoneme conversion, which involves the systematic mapping of visual letter forms (graphemes) onto their corresponding speech sounds (phonemes) [29]. The postulated disruption to lexical processing in PCA may lead these individuals to rely disproportionately on the slower non-lexical (i.e., grapheme-to-phoneme conversion) route. This over-reliance may lead to errors common to the disruption of the lexical pathway, including word-length effects, letter-by-letter reading, and phonologically plausible errors when spelling irregular words.

Our primary aim in this descriptive study was to characterize deficits observed in reading and spelling abilities within our Massachusetts General Hospital (MGH) PCA cohort who are largely at a mild stage of illness severity. We hypothesize that we will observe reading impairment secondary to visual processing dysfunction in the form of skipped lines and letter transpositions, as well as spelling impairment characterized by orthographic dysfunction. Understanding the specific characteristics of these deficits is important for developing interventions aimed at improving or preserving functional literacy in PCA patients, as traditional language-based strategies may prove ineffective in the context of prominent visual impairments. We hope that evaluating these abilities in detail will contribute useful information to the comprehensive characterization of patients with PCA syndrome and will assist with diagnostic characterization and inform targeted treatment approaches for these individuals.

## 2. Methods

### 2.1. Participant Characteristics

Data for this study were obtained from 23 participants who met the diagnostic criteria for PCA in the Massachusetts General Hospital Frontotemporal Disorders Unit PCA program [30,31]. Demographic and clinical characteristics are presented in Table 1. All but one participant were born in the United States and reported English to be their first and only language. The last participant (referenced as Case B and Case F in their respective results sections) reported that she learned English concurrently with her other native language, Telugu. All participants received a standard clinical evaluation comprising a neurological and psychiatric history and exam and structured informant interviews following the Clinical Dementia Rating (CDR) protocol, resulting in a global CDR score and a sum-of-boxes score (CDR-SB), as well as a comprehensive speech and language evaluation. As part of the CDR supplemental rating system, a Language box score was also rated. Each participant was also assessed with the Montreal Cognitive Assessment (MoCA) screening tool [32] conducted by a neurologist or neuropsychologist to determine the global level of cognitive impairment. For each patient, clinical diagnostic formulation was performed through consensus conference, with each patient being classified based on all clinical information as having mild cognitive impairment or dementia (global clinical status), and then each patient’s cognitive–behavioral syndrome being diagnosed according to standard diagnostic criteria [33,34]. All 23 participants met core diagnostic criteria for “PCA” [1,35] and further met criteria for “PCA-pure” [1]. Of the total sample, 21 participants had imaging (amyloid PET) or cerebrospinal fluid (CSF) biomarker status consistent with AD pathology. Amyloid status was unknown for one participant, and negative for the remaining participant. Of the patients who underwent amyloid PET imaging, amyloid positivity was determined by a combination of visual read and mean amyloid PET signal extracted from a cortical composite region of interest according to previously published procedures [36]. Other patients underwent CSF sampling, with results indicating abnormally low levels of CSF amyloid and abnormally high levels [37]. Individuals were excluded from this study if they had a primary psychiatric or other neurologic disorder including major cerebrovascular infarct or stroke, seizure, brain tumor, hydrocephalus, multiple sclerosis, HIV-associated cognitive impairment, or acute encephalopathy. Additionally, the presence of disorders of auditory perception and speech intelligibility (e.g., motor speech) were exclusionary criteria for participants in this study. This work was carried out in accordance with The Code of Ethics of the World Medical Association (Declaration of Helsinki) for experiments involving humans. All participants and their informants/caregivers provided informed consent in accordance with the protocol approved by the Mass General Brigham Human Research Committee Institutional Review Board in Boston, Massachusetts.

### 2.2. Speech and Language Evaluation

To comprehensively characterize the speech and language profiles of these participants, we utilized the Progressive Aphasia Severity Scale (PASS) [38,39], which has been reported to show excellent inter-rater reliability with high Intraclass Correlation Coefficients (ICC) of over 0.9 for ratings in each domain [38]. We developed this tool to expand upon the Clinical Dementia Rating (CDR) [40] supplemental language box score. The PASS is a structured clinical instrument used to describe the level of impairment in ten distinct domains of speech and language on a scale from 0 (normal) to 3 (severe): Articulation, Fluency, Syntax/Grammar, Word Retrieval, Repetition, Auditory Comprehension, Single Word Comprehension, Reading, Writing, and Functional Communication (Table 2). PASS ratings are as follows: 0 = clinically normal, 0.5 = questionable/very mild impairment, 1 = mild impairment, 2 = moderate impairment, 3 = severe impairment. Ratings were made using the clinician’s judgment of the overall level of impairment in each domain, based on information from a structured interview with the patient and partner, and from performance on the speech and language assessment. Although the PASS was developed for evaluating language impairment in primary progressive aphasia, it was included here to provide a structured assessment of language function in participants with PCA. This allowed us to characterize any accompanying linguistic deficits (e.g., in naming, fluency, or comprehension) and to contextualize reading and spelling difficulties within each participant’s overall language profile.

In the current study, we analyzed performance on tests of reading and spelling. The Western Aphasia Battery (WAB) [41] Reading Comprehension subtest was used to assess reading comprehension. In this task, the participant is presented with eight test sentences and instructed to select the most appropriate missing word from a provided list to complete each sentence. Length and difficulty increase as the task advances. The ability to follow written commands was assessed through the WAB Reading Commands subtest, with some participants completing an informal commands task as a supplement to the WAB subtest. The WAB, in general, has high internal consistency, inter- and intra-rater reliability, and test–retest reliability [42]. Furthermore, there is strong evidence that the scores derived from the videoconference administration of these tests showed high concordance, supporting the use of telehealth administration of the WAB in videoconferencing [43]. During these tasks, the participant was instructed to read a command aloud and execute the specified action. The complexity of the command sentence increases as the task advances. To evaluate spelling abilities, the Boston Diagnostic Aphasia Examination (BDAE) [44] spelling subtest was administered, targeting participants’ capacity to accurately spell both regular and irregular words that the clinician reads aloud. To our knowledge, no studies formally evaluating the internal consistency of this widely used gold standard evaluation of language have been published. The assessments were conducted in in-person or virtual settings using Zoom technology, with 16 participants evaluated face-to-face and 7 assessed remotely. Both methods of administration allowed for direct observation of nonverbal behaviors, such as visual strain or task-specific challenges. Virtual sessions utilized screen-sharing features to present stimuli and HIPAA approved video conferencing tools for monitoring participant responses. These approaches collectively ensured that the data obtained reflected a thorough evaluation of language abilities across different settings.

### 2.3. Visuospatial Impairment Rating (VIR) Scale

We developed the Visuospatial Impairment Rating (VIR) scale [45] to characterize the clinical severity of patients in the visual cognitive domain. As this is a tool that is used purely descriptively and has not yet been evaluated for psychometric properties, we do not have any reports of reliability statistics. The format of the rating scale is similar to a Clinical Dementia Rating (CDR) scale [40]. The VIR is designed to capture the overall level of visuospatial impairment as it relates to cerebral dysfunction, such as visual object agnosia, prosopagnosia, visuoperceptual impairment, simultanagnosia, hemispatial neglect, hemianopsia, or cortical blindness, that are not attributed to primary ocular or extra-ocular movement dysfunction or cognitive impairment in non-visual domains. The VIR was rated by integrating information about visuospatial symptoms in daily life and visuoperceptual and visual–cognitive abnormalities on examination, without knowledge of neuropsychological test performance. VIRs of all participants in this study, similar to CDRs, were conducted by a neurologist or neuropsychologist, and were based on a semi-structured patient and informant interview and a neurologic examination of the patient that specifically includes evaluation of cortical visual dysfunction. VIRs were as follows: 0 = clinically normal, 0.5 = questionable/very mild impairment, 1 = mild impairment, 2 = moderate impairment, 3 = severe impairment, and reported in Table 1 along with CDR scores.

### 2.4. Mixed-Methods Data Analysis

Total summary scores were calculated for each of the tests used to evaluate reading (WAB Reading Comprehension, WAB Reading Commands) and spelling (BDAE Spelling subtest). Mean scores and standard deviations were reported for those who completed tasks in a standardized fashion. In light of the fact that scores on the tests of reading and spelling we focused on were not normally distributed (all variables significantly deviated from normality according to the Shapiro–Wilk test (all *p* < 0.05)), Spearman’s rho correlation coefficients (r_s_) were calculated to determine relationships between measures of interest. To determine the association between reading and spelling performance and global cognitive and functional impairment, Spearman’s correlations were conducted between the total scores on these three tasks and a global measure of cognition (MoCA score), global functional impairment (CDR-SB), overall language functional impairment (PASS), and visuospatial functional impairment (VIR). We also conducted Spearman’s correlations to determine the association between functional language impairment (PASS) and overall global cognition (MoCA), clinical severity (CDR-SB) and visuospatial impairment (VIR). These statistical analyses were conducted in IBM SPSS Version 24.0 (Armonk, NY, USA). Qualitative notes are also reported on task approach and performance, with examples of spelling errors presented in Table 3. Case examples showcasing the types of errors observed across tests are also provided.

## 3. Results

### 3.1. Clinical and Demographic Characteristics

Twenty-three individuals diagnosed clinically with the PCA syndrome were included in this study. The vast majority of these individuals (21 out of 23) were rated at the stage of mild cognitive impairment (CDR 0.5) or very mild dementia (CDR 1), with only two individuals at the stage of moderate dementia (CDR 2). Scores on the CDR Supplemental Language box score indicated that this PCA cohort as a whole was either very mildly impaired (*n* = 10 rated as 0.5) or mildly impaired (*n* = 13 rated as 1) in the language domain. In comparison, the scores on the VIR reflected the greatest area of severity being the visuospatial domain—only 1 out of 23 individuals was rated as very mildly impaired (CDR 0.5), 17 were rated as mildly impaired (CDR 1), and 5 were rated as moderately impaired (CDR 2). See Table 1 for complete sample characteristics. On average, the cohort was otherwise racially homogenous (96% White). On average, these participants were 66.0 ± 8.9 years of age and highly educated (17.0 ± 2.0 years), with 61% of the group being female and 96% being right-handed.

### 3.2. Characterizing Language with the PASS

The PASS was used to rate language skills as it provides a thorough analysis of language functioning across several key domains. Scores were derived from informant responses and patient abilities, integrated through clinician judgment, in ten discrete domains. See Table 2 for complete sample PASS subdomain scores. We found that certain language functions were disproportionately impacted in this cohort, while others remained relatively spared. Specifically, speech articulation, language fluency, and syntax/grammar were generally spared, indicating that participants did not exhibit significant apraxia of speech or agrammatism. Auditory comprehension showed only mild impairment, while single-word comprehension was completely intact, suggesting that participants had no difficulty understanding isolated words. In contrast, we found that participants experienced moderate difficulty in the domains of word retrieval, reading, and writing. Overall functional communication was also moderately impacted, reflecting challenges in day-to-day communication and language use in the impacted areas. The PASS was not associated with the CDR-SB (r_s_ = 0.27, *p* = 0.23) or VIR scores (r_s_ = −0.11, *p* = 0.63), indicating some level of specificity in capturing clinical severity in the language domain separately from global clinical severity or clinical severity in the visuospatial domain. There was trend-level association with the MoCA (r_s_ = −0.43, 0.05).

### 3.3. Assessment of Reading Skills

Our evaluation of reading abilities revealed a pattern of deficits consistent with the hallmark visual processing impairments in PCA. A comprehensive table of performance across all tests and clinical scales is presented in Supplementary Materials Table S1. Performance on the WAB Reading Comprehension test varied substantially, and was not associated with global cognitive impairment (MoCA; r_s_ = 0.37, *p* = 0.11). Even very mildly impaired individuals who scored well on this test were observed to have difficulty following the lines of text and required prompting and scaffolding with verbal cues. Some participants reported a high level of visual strain when asked to read. In contrast, performance on the Reading Command test was significantly associated with global cognitive severity (MoCA: r_s_ = 0.50, *p* = 0.03).

#### 3.3.1. WAB Reading Comprehension

Out of the 23 participants who underwent the WAB Reading Comprehension test, 20 individuals were able to complete this test with standardized administration. In this group, the total score out of 40 was 38.6 (mean) ± 0.072 (SD), indicating a strong performance. Sixteen of these individuals demonstrated intact decoding and reading comprehension abilities, but the remaining four individuals had difficulty visually scanning lines to read independently (e.g., Case A). In these individuals, we also observed difficulty primarily on the paragraph-level items of the task, with several decoding and comprehension errors and a slowed reading pace. For the last 3 participants, the task was discontinued per participant request due to the level of visual demand (e.g., Case B) or non-standardized administration was performed. (e.g., Case C). Across these participants, the observed difficulties were associated with varying visual impairments. Case A exhibited attentional neglect for the left visual field, leading to missed line onsets. Case B demonstrated impaired visual attention and scanning, resulting in misreading of multi-line text. Case C demonstrated striking visual agnosia as well as visual attention deficits with largely intact comprehension. Regarding the association between performance on the WAB Reading Comprehension test and clinical severity, scores on this test trended toward a significant association with CDR-SB (r_s_ = −0.43, *p* = 0.05), but were not associated with PASS ratings (r_s_ = −0.30, *p* = 0.19), or VIR scores (r_s_ = −0.15, *p* = 0.53).

##### Case Examples

**Case A:** A 77-year-old Caucasian female with a global CDR of 0.5 and a VIR score of 1 achieved ceiling accuracy (100%) on reading comprehension tasks. Although her performance was strong, she occasionally omitted the initial words on the second line of text during the WAB Reading Comprehension task, prompting rereading for accurate comprehension. The observed omissions are consistent with attentional neglect for the left visual field, leading to missed line onsets during multi-line reading. This performance pattern suggests preserved contextual reading comprehension skills despite visual scanning difficulty that is a hallmark characteristic of PCA syndrome.

**Case B:** A 57-year-old Asian female with a global CDR of 1 and a VIR score of 2 reported visual strain and difficulty reading multiple lines of text. Her reading was slow and involved repetition and reading errors (read “busy” for “bus” and “train” for “rain”). Her reading errors suggest that she was relying on context and familiar patterns to interpret words, likely as a way to compensate for difficulties accurately perceiving visual information. The task was ultimately discontinued due to difficulty. Her visuospatial functioning was characterized primarily by moderate-to-severe impairment in visual attention and visual object identification.

**Case C:** A 74-year-old Caucasian female with a global CDR of 1 and a VIR score of 2 demonstrated severe visual processing challenges while reading, requiring the task to be discontinued. In this case, the examiner was required to read the sentences and choices within the task aloud, shifting the task from a reading comprehension assessment to an auditory comprehension task. The participant performed well with this modification, indicating intact auditory comprehension in the context of marked alexia. Her visuospatial functioning was characterized primarily by moderate-to-severe impairment in visual object identification and secondarily by impairment in visual attention.

#### 3.3.2. Reading Commands

We evaluated participants with the WAB Reading Commands task, and in some cases, an Informal Commands task. A total of 20 participants completed these tests with standardized administration; the average score out of 20 was 18.5 (mean) ± 0.18 (SD). Together, these assessments provided a comprehensive evaluation of participants’ ability to read and execute written instructions. Of the 20 participants who attempted these tests, 15 completed the task accurately with intact reading and action completion (e.g., Case D). Two participants made minor errors in either partial action completion or personalizing the read-aloud instructions (e.g., reading “raise my hand” instead of “raise your hand”). For the remaining three participants, errors increased with multi-line instructions (e.g., Case E). One participant demonstrated a high degree of difficulty with the visual demand of this reading task, complaining of visual discomfort and blurring, leading to discontinuation (e.g., Case F). Performance on this test was not associated with CDR-SB (r_s_ = −0.10, *p* = 0.67) or VIR scores (r_s_ = 0.37, *p* = 0.11), but was moderately associated with PASS scores (r_s_ = −0.52, *p* = 0.02).

##### Case Examples

**Case D:** A 58-year-old Caucasian male with a global CDR of 1 and a VIR score of 2 successfully read all single and multi-line commands while demonstrating precise execution of the corresponding actions. His visuospatial impairment was characterized as a deficit in space perception but he was noted to demonstrate intact visual object identification.

**Case E:** A 62-year-old Caucasian female with a global CDR of 1 and a VIR score of 2 did not read or perform actions for the final three longer items within the task, indicating vulnerability to length and complexity during reading tasks. She was noted to have significant visual object identification difficulty as well as mild comprehension impairment.

**Case F:** A 57-year-old Asian female with a global CDR of 1 and a VIR score of 2 struggled with items that included multi-line text and neglected reading the right-sided portions of the sentences, indicating visual neglect. Complaints of discomfort and blurring were documented. The examiner attempted to use contrast as a scaffold by highlighting the black text in yellow, though this provided limited benefit. Severity of visual processing deficits, including neglect and reduced contrast sensitivity, necessitated task discontinuation.

### 3.4. Assessment of Spelling Skills

Our evaluation of spelling revealed that errors were common in this sample; performance was not associated with global cognitive impairment (MoCA; r_s_ = 0.26, *p* = 0.32). Spelling was assessed in 19 participants using the BDAE Spelling subtest, with the remaining four participants not completing the task due to visuoperceptual dysfunction. This test evaluates written language abilities by measuring the accuracy of spelling regular and irregular words written to dictation, intended to provide insight into phonological and orthographic processing. Of the 17 participants who completed this test with standardized administration (one individual requested to spell all words orally instead of writing them), the average score was 10.2 (mean) ± 0.15 (SD) out of 12, indicating mild impairment. Only five participants spelled all words accurately (e.g., Case G), and six participants made a single error on an irregular word (the most common being “yacht”). The remaining six participants’ spelling performance revealed marked difficulty with spelling irregular and multisyllabic words, with errors characterized by regularization (e.g., “rane” for “reign”), reliance on phonetic approximations (e.g., “quire” for “choir), substitutions (e.g., “yaght” for “yacht”), and transpositions (e.g., “yatch” for “yacht”—e.g., Case H). Visual deficits further impacted letter formation, leading to inconsistent letter formation and omissions (e.g., missing the crossbar on “h” in “choir”). One moderately impaired participant demonstrated difficulty with written spelling and requested to transition to oral spelling mid-task, though regularization errors in spelling persisted with oral spelling (e.g., Case I—rane for reign). Overall, while the spelling errors observed in this cohort reflected orthographic and phonological processing difficulties, visual–orthographic challenges specifically impacting letter formation and consistency also contributed to handwriting errors in a subset of participants. Refer to Table 3 for observed error variants. Scores on this spelling test were not associated with CDR-SB ratings (r_s_ = 0.05, *p* = 0.83), PASS ratings (r_s_ = −0.36, *p* = 0.14), or VIR scores (r_s_ = 0.27, *p* = 0.27).

#### Case Examples

**Case G:** A 62-year-old Caucasian female with a global CDR of 1 and a VIR score of 2 spelled all words accurately, indicating intact visual processing of written language, lexical retrieval and orthographic memory. Her visuospatial cognitive profile was notable for simultanagnosia but she had intact single object identification.

**Case H:** A 75-year-old Caucasian female with a global CDR of 0.5 and a VIR score of 1 exhibited pervasive difficulties with irregular words, producing errors such as “arartment” for “apartment” and “ghoir” for “choir.” Handwriting attempts were marked by repeated revisions, distortions, and inconsistent letter formations (e.g., missing bar for the letter /H/). These patterns reflect visual–orthographic challenges, impacting her ability to perceive and write letter shapes accurately. Her visuospatial cognitive profile was notable for single object identification impairment as well as visual attention deficit.

**Case I:** A 69-year-old Caucasian male, with a global CDR of 0.5 and a VIR score of 1, transitioned to oral spelling when visual deficits impacted handwriting. This participant had particular trouble writing the letters /G, A, and N/ reflecting possible visual–orthographic challenges in producing letter shapes. This oral compensatory approach, intended to bypass visual processing challenges, revealed the continued presence of regularization errors and a reliance on phonetic approximation when spelling words verbally (“rane/reign” “qur/choir” “yhach/yacht”), indicating that the underlying spelling difficulties may extended beyond visual impairments. His cognitive profile indicated a primary impairment in agraphia in the absence of other visual perceptual impairment.

## 4. Discussion

We sought to shine a light on the heterogeneity of reading and spelling abilities in PCA by characterizing performance on tests of reading comprehension, reading commands, and the spelling of regular and irregular words. As a result of dysfunctional occipital–temporal and lateral parietal cortices in PCA, reading and spelling are highly vulnerable skills in this population [11,46]. Despite this, reading and spelling are rarely formally evaluated in PCA, perhaps in part because it is challenging for patients to complete standardized evaluations due to the heavy visual demand inherent in these tests. Recent studies indicate that reading and spelling difficulties in PCA may arise from a mix of visual and non-visual (e.g., language) deficits [47]. When evaluating reading and spelling in individuals with PCA, it is crucial to incorporate multimodal assessment strategies that account for the complex interplay between visual, phonological, and lexical processing. It is also important to qualitatively describe and interpret errors, as this will have direct implications for devising treatment strategies. Here, we present methodology to characterize reading and spelling abilities in patients with PCA using a mixed quantitative and qualitative approach.

### 4.1. Heterogeneity in Reading

In our evaluation of reading, we found that the majority of participants who could complete standardized evaluations performed at ceiling level, indicating largely intact decoding and comprehension abilities. However, a subset of participants exhibited challenges related to visual scanning on the lengthier passages of the WAB Reading Comprehension task which required reading multiple lines of text. These participants required redirection to scaffold their visual attention. The observed errors, including omissions, misreading, and reliance on auditory support or modifications in visual contrast, underscore the role of context-dependent visual processing deficits in understanding reading vulnerabilities in this cohort. Interestingly, we observed that individuals who had more difficulty with reading performance had marked visual agnosia (with or without visual attention deficits) while those who had less difficulty with this task had intact visual object identification but other visual cognitive deficits (e.g., simultanagnosia or space perception deficit) characteristic of PCA. These findings add to the growing literature highlighting marked heterogeneity in reading impairments among individuals with PCA particularly when reading tasks involve increased perceptual demands [17,19]. This pattern of reading performance is generally attributed to impaired visual attention and visual crowding effects prevalent in PCA [14,15,19]. It has also been previously reported that individuals with PCA have difficulty maintaining their place while reading, including trouble tracking lines of text, neglect and word order errors, and greater susceptibility to visual discomfort such as sensitivity to glare [14,17]. As mentioned previously, the DRC model provides a helpful lens for interpreting these findings. Within this framework, the observed errors could be understood as arising from impairment at the early stage of mapping visual input to orthographic representations, leading to reliance on slower and less efficient sub-lexical routes. This approach may have resulted in the observed errors, including neglect dyslexia, misreading, and slowed reading pace [48]. We extended previous observations of these difficulties in PCA by reporting this to be the case even in individuals at milder stages of clinical severity. Our selected case examples further underscore the heterogeneity of reading impairments in PCA, expanding upon prior case reports [6,49] by describing the nature of reading challenges across a clinical severity spectrum of very mild to moderate impairment.

### 4.2. Heterogeneity in Spelling

Our cohort also demonstrated a wide range of spelling abilities, with some individuals demonstrating preserved orthographic skills and others displaying pronounced deficits, particularly with irregular and multisyllabic words. While a subset of participants performed well, the majority made varying types of errors largely characterized by phonetic approximations, regularization, and letter substitutions or transpositions. These observations are consistent with prior reports describing heterogenous spelling errors in individuals with PCA that frequently reflect vulnerability in graphemic output buffer and phonological encoding [6,8]. Letter-level errors and disproportionate difficulty with spelling longer words are thought to be hallmarks of graphemic buffer dysfunction which is linked to left parietal cortex dysfunction, a region important for working memory and orthographic processing [50]. Neuroimaging findings also implicate inferior temporal regions in the progression of spelling impairments in individuals with PCA [46]. Consistent with this neuroanatomical observation, we observed those individuals who had greater difficulty completing this spelling test were also observed to have significant visual agnosia (a ventral visual stream function) with or without visual attention deficits. While visual processing impairment likely contributed to spelling errors in the majority of participants leading to inconsistent letter formation, omissions, and revisions consistent with dysgraphia, one participant transitioned to oral spelling to compensate for visual strain and yet continued to produce phonological and regularization errors. This suggests that spelling difficulties may extend beyond visual constraints to reflect underlying language impairments in some individuals with PCA. Difficulty in spelling irregular words may also be better understood through the framework of the DRC model. Spelling irregular words typically relies on the lexical route of the model to access familiar stored words in the orthographic lexicon [29,48]. According to the model, regularization errors arise when the non-lexical/grapheme-to-phoneme conversion route is used for spelling irregular words, bypassing the orthographic lexicon and instead applying standard grapheme-to-phoneme conversion rules. It is likely that in PCA, disruption in visual–orthographic processing may limit efficient access to the lexical route, forcing greater reliance on the non-lexical route. This perspective is consistent with prior reports of phonological errors in the speech of individuals with PCA, as well as the presence of surface dysgraphia, phonological dysgraphia, or broader features of more central dysgraphia during oral spelling tasks in these individuals [8,46]. It is important to acknowledge that such errors may also arise from other deficits, such as challenges in phonological processing, auditory perception, or speech intelligibility, which may be prevalent in older adults (though auditory and motor speech deficits were exclusionary criteria for this study).

### 4.3. Reading and Spelling Errors Are Not Primarily a Function of Global Clinical Severity

Performance on the reading comprehension and spelling tests was independent of global cognitive impairment (MoCA), overall clinical severity (CDR-SB), functional language impairment (PASS), and visuospatial functional impairment (VIR), indicating that alexia and spelling dysfunction in this syndrome is not solely observed as a function of global illness severity. Consistent with this, we observed that several patients with very mild cognitive impairment (CDR of 0.5) made spelling errors and demonstrated difficulty with visual scanning during reading tasks. Though performance on Reading Commands was not significantly associated with VIR scores, we did observe that performance on this task was positively associated with MoCA scores and negatively associated with PASS scores. These findings should be interpreted with caution, as lower Reading Commands scores in some participants were driven by visual processing difficulties and comprehension vulnerability rather than true deficits in command execution, perhaps suggesting a combination of linguistic and visual processing impairment associated with the completion of this task. Limitations of utilizing this task to determine reading ability are discussed below. Of note, MoCA and PASS scores were associated with each other at a trend level, indicating some shared variance and underlying functional language difficulty contributing to both scores.

### 4.4. Limitations

This study had several limitations that should be considered when interpreting the findings. Testing this population sometimes necessitated non-standardized test administration, which could introduce variability in the results. To address this, we emphasized qualitative descriptors in our characterization of reading and spelling performance in this cohort to achieve our aim of better understanding these aspects of cognitive dysfunction in PCA. We also aimed to evaluate reading comprehension and ability to follow written commands in a population characterized by visual processing deficits. As such, for a small subset of participants, performance on the WAB Reading Comprehension and Reading Commands tasks was influenced by visual processing difficulties, such as difficulty tracking lines of text and visual fatigue. Given this, we acknowledge that these tasks may have reduced validity for purely assessing reading and command execution in individuals with pronounced visuospatial impairments due to PCA and should be utilized with this in mind. Although standard reading assessments are inherently dependent on visual processing, future studies should consider exploring and incorporating assessment methods that reduce visual demand. Another limitation pertains to the generalizability of these findings in light of the homogenous demographic characteristics of this sample regarding age range, high level of education, race (mostly Caucasian), and overall mild level of clinical severity (mostly CDR 0.5 or 1). Further work is needed to replicate these findings in diverse populations, including low- and middle-income populations with a range of formal educational attainment. Last, the mix of virtual and in-person evaluations may also have introduced variability, as test conditions differ between these modalities. However, it is reassuring that studies of testing efficacy have shown that the results from virtual assessments of language are largely comparable to those obtained in person [51].

### 4.5. Implications and Future Directions

Taken together, these results suggest a phenotypic heterogeneity in the syndromic presentation of PCA, with some individuals having particular difficulty with reading and spelling while others do not. These results warrant further investigation and may offer contributing evidence to hypothesized subtypes of PCA that are just beginning to be explored in the literature [1,52].

This work also has practical implications for speech–language pathologists and other clinicians. Given the intersection of visual and language impairments in PCA, speech–language pathologists, neuropsychologists, and occupational therapists are well-positioned to contribute to comprehensive treatment by helping to design adaptive communication strategies and tailoring interventions that accommodate both language and visual processing challenges as part of multidisciplinary assessment that also includes evaluation of visuoperceptual and oculomotor deficits. Tailoring interventions to address the specific challenges these patients face in reading, spelling, and other vital language domains (e.g., with a focus on vision rehabilitation or linguistic support) may lead to more effective and individualized therapeutic approaches, ultimately improving quality of life for individuals living with PCA. Ultimately, systematic evaluation with tools designed specifically for use in PCA syndrome and in larger cohorts will be a helpful next step in better understanding the heterogeneity of reading and spelling abilities in PCA. Another useful avenue of investigation would be the development of functional neuroimaging paradigms that directly probe reading and spelling in individuals with PCA, as this would help clarify the neural mechanisms underlying the varied deficits observed in this study.

## 5. Conclusions

This descriptive study utilizing a mixed quantitative and qualitative approach found heterogeneous profiles of reading and spelling abilities in individuals living with PCA syndrome, characterized by visual scanning errors (e.g., neglect dyslexia) and spelling errors (e.g., surface dysgraphia), reflecting both visual–perceptual and orthographic–linguistic breakdowns. These findings add to our current characterization of the PCA syndrome by offering a more detailed understanding of reading and spelling abilities, skills that are often overshadowed by the primary focus on visuospatial deficits. By investigating these abilities with a mixed quantitative and qualitative methodology, this paper underscores the importance of considering both visual and lexical deficits as contributing to reading and spelling difficulties.

## Figures and Tables

**Table 1:** Clinical characteristics. Means and standard deviations (SDs) are reported for continuous variables. MoCA = Montreal Cognitive Assessment. CDR = Clinical Dementia Rating Scale. VIR = Visuospatial Impairment Rating scale.

Clinical/Demographic Characteristic	*n* = 23
Age (years)	66.0 ± 8.9
Sex (M/F)	9/14
Education (years)	17.0 ± 2.0
Race	White (*n* = 22)
	Asian (*n* = 1)
Handedness	Right-handed (*n* = 22)
	Ambidextrous (*n* = 1)
MoCA (/30)	18.9 ± 5.4
CDR-Global	CDR 0.5 (*n* = 13)
	CDR 1 (*n* = 8)
	CDR 2 (*n* = 2)
CDR-SB	4.0 ± 2.8
CDR-Language	CDR-L box score 0.5 (*n* = 10)
	CDR-L box score 1 (*n* = 13)
VIR	VIR 0.5 (*n* = 1)
	VIR 1 (*n* = 17)
	VIR 2 (*n* = 5)

**Table 2:** Progressive Aphasia Severity Scale (PASS). Clinical ratings of impairment were conducted for 10 domains of speech and language. Means and standard deviations of PASS box scores are reported. See Sapolsky et al. 2014 [39] for details on the PASS.

PASS Domains	N = 23
Articulation	0.02 ± 0.10
Fluency	0.11 ± 0.21
Syntax/Grammar	0.09 ± 0.19
Word Retrieval	0.54 ± 0.21
Repetition	0.22 ± 0.25
Auditory Comprehension	0.20 ± 0.36
Single Word Comprehension	0.00 ± 0.00
Reading	0.50 ± 0.85
Writing	0.89 ± 0.99
Functional Communication	0.17 ± 0.24

**Table 3:** Spelling error variants. Each example represents responses by participants that deviated from the accurate spelling of the target word.

Target Word	Error Variants
**Apartment**	arpment, arartment, aprament
**Reign**	reine, rein, raine, rane (oral spelling), rain
**Choir**	chour, quire, quiarne, qur (oral spelling), coire, ghoir (crossbar missing on “h”), quier
**Yacht**	yatch, yaght (repeated multiple times), yat, yaht, yochk, yhach (oral spelling), youdt
**Cough**	cauf
**Knife**	nife (oral spelling), knive
**Nation**	naticon, natin

## Data Availability

The data presented in this study are available on request from the corresponding author due to reasons related to confidentiality and protecting privacy.

## Supplementary Materials

The following supporting information can be downloaded at: https://www.mdpi.com/article/10.3390/brainsci15111154/s1, Figure S1: Associations between cognitive and clinical severity with tests of reading and spelling; Table S1: Individual-level performance scores on clinical measures and tests of reading and spelling.

## References

[1] Crutch S.J., Schott J.M., Rabinovici G.D., Murray M., Snowden J.S., van der Flier W.M., Dickerson B.C., Vandenberghe R., Ahmed S., Bak T.H. (2017). Consensus classification of posterior cortical atrophy. Alzheimer’s Dement..

[2] Mendez M.F., Ghajarania M., Perryman K.M. (2002). Posterior cortical atrophy: Clinical characteristics and differences compared to Alzheimer’s disease. Dement. Geriatr. Cogn. Disord..

[3] Benson D.F., Davis R.J., Snyder B.D. (1988). Posterior cortical atrophy. Arch. Neurol..

[4] Yerstein O., Parand L., Liang L.J., Isaac A., Mendez M.F. (2021). Benson’s Disease or Posterior Cortical Atrophy, Revisited. J. Alzheimer’s Dis..

[5] Chapleau M., La Joie R., Yong K., Agosta F., Allen I.E., Apostolova L., Best J., Boon B.D.C., Crutch S., Filippi M. (2024). Demographic, clinical, biomarker, and neuropathological correlates of posterior cortical atrophy: An international cohort study and individual participant data meta-analysis. Lancet Neurol..

[6] Brodeur C., Belley E., Deschenes L.M., Enriquez-Rosas A., Hubert M., Guimond A., Bilodeau J., Soucy J.P., Macoir J. (2022). Primary and Secondary Progressive Aphasia in Posterior Cortical Atrophy. Life.

[7] Magnin E., Sylvestre G., Lenoir F., Dariel E., Bonnet L., Chopard G., Tio G., Hidalgo J., Ferreira S., Mertz C. (2013). Logopenic syndrome in posterior cortical atrophy. J. Neurol..

[8] Tetzloff K.A., Duffy J.R., Strand E.A., Machulda M.M., Schwarz C.G., Senjem M.L., Jack C.R., Josephs K.A., Whitwell J.L. (2021). Phonological Errors in Posterior Cortical Atrophy. Dement. Geriatr. Cogn. Disord..

[9] Putcha D., Dickerson B.C., Brickhouse M., Johnson K.A., Sperling R.A., Papp K.V. (2020). Word retrieval across the biomarker-confirmed Alzheimer’s disease syndromic spectrum. Neuropsychologia.

[10] Putcha D., Eustace A., Carvalho N., Wong B., Quimby M., Dickerson B.C. (2024). Auditory naming is impaired in posterior cortical atrophy and early-onset Alzheimer’s disease. Front. Neurosci..

[11] Crutch S.J., Lehmann M., Warren J.D., Rohrer J.D. (2013). The language profile of posterior cortical atrophy. J. Neurol. Neurosurg. Psychiatry.

[12] Rezaii N., Hochberg D., Quimby M., Wong B., McGinnis S., Dickerson B.C., Putcha D. (2024). Language uncovers visuospatial dysfunction in posterior cortical atrophy: A natural language processing approach. Front. Neurosci..

[13] McMonagle P., Deering F., Berliner Y., Kertesz A. (2006). The cognitive profile of posterior cortical atrophy. Neurology.

[14] Crutch S.J., Lehmann M., Schott J.M., Rabinovici G.D., Rossor M.N., Fox N.C. (2012). Posterior cortical atrophy. Lancet Neurol..

[15] Pavisic I.M., Yong K.X.X., Primativo S., Crutch S.J., Suarez Gonzalez A. (2019). Unusual Pattern of Reading Errors in a Patient with Posterior Cortical Atrophy. Case Rep. Neurol..

[16] Crutch S.J., Lehmann M., Gorgoraptis N., Kaski D., Ryan N., Husain M., Warrington E.K. (2011). Abnormal visual phenomena in posterior cortical atrophy. Neurocase.

[17] Yong K.X., Rajdev K., Shakespeare T.J., Leff A.P., Crutch S.J. (2015). Facilitating text reading in posterior cortical atrophy. Neurology.

[18] Suarez-Gonzalez A., Ocal D., Pavisic I., Peacock A., Naessens M., Ahmed S., Butler C.R., Leff A.P., Yong K.X.X., Crutch S.J. (2019). ReadClear: An Assistive Reading Tool for People Living with Posterior Cortical Atrophy. J. Alzheimer’s Dis..

[19] Yong K.X., Shakespeare T.J., Cash D., Henley S.M., Nicholas J.M., Ridgway G.R., Golden H.L., Warrington E.K., Carton A.M., Kaski D. (2014). Prominent effects and neural correlates of visual crowding in a neurodegenerative disease population. Brain.

[20] Li J., Wu L., Tang Y., Zhou A., Wang F., Xing Y., Jia J. (2018). Differentiation of neuropsychological features between posterior cortical atrophy and early onset Alzheimer’s disease. BMC Neurol..

[21] Epelbaum S., Pinel P., Gaillard R., Delmaire C., Perrin M., Dupont S., Dehaene S., Cohen L. (2008). Pure alexia as a disconnection syndrome: New diffusion imaging evidence for an old concept. Cortex.

[22] Mummery C.J., Patterson K., Wise R.J., Vandenberghe R., Price C.J., Hodges J.R. (1999). Disrupted temporal lobe connections in semantic dementia. Brain.

[23] Wilson S.M., Rising K., Stib M.T., Rapcsak S.Z., Beeson P.M. (2013). Dysfunctional visual word form processing in progressive alexia. Brain.

[24] Best J., Chapleau M., Rabinovici G.D. (2023). Posterior cortical atrophy: Clinical, neuroimaging, and neuropathological features. Expert. Rev. Neurother..

[25] Agosta F., Mandic-Stojmenovic G., Canu E., Stojkovic T., Imperiale F., Caso F., Stefanova E., Copetti M., Kostic V.S., Filippi M. (2018). Functional and structural brain networks in posterior cortical atrophy: A two-centre multiparametric MRI study. Neuroimage Clin..

[26] Migliaccio R., Agosta F., Scola E., Magnani G., Cappa S.F., Pagani E., Canu E., Comi G., Falini A., Gorno-Tempini M.L. (2012). Ventral and dorsal visual streams in posterior cortical atrophy: A DT MRI study. Neurobiol. Aging.

[27] Grainger J. (2018). Orthographic processing: A ‘mid-level’ vision of reading: The 44th Sir Frederic Bartlett Lecture. Q. J. Exp. Psychol..

[28] Wang F., Maurer U. (2020). Interaction of top-down category-level expectation and bottom-up sensory input in early stages of visual-orthographic processing. Neuropsychologia.

[29] Coltheart M., Rastle K., Perry C., Langdon R., Ziegler J. (2001). DRC: A dual route cascaded model of visual word recognition and reading aloud. Psychol. Rev..

[30] Putcha D., McGinnis S.M., Brickhouse M., Wong B., Sherman J.C., Dickerson B.C. (2018). Executive dysfunction contributes to verbal encoding and retrieval deficits in posterior cortical atrophy. Cortex.

[31] Wong B., Lucente D., MacLean J., Padmanabhan J., Quimby M., Brandt K.D., Putcha D., Sherman J.C., Frosch M.P., McGinnis S. (2019). Diagnostic evaluation and monitoring of patients with Posterior Cortical Atrophy. Neurodegener. Dis. Manag..

[32] Nasreddine Z.S., Phillips N.A., Bedirian V., Charbonneau S., Whitehead V., Collin I., Cummings J.L., Chertkow H. (2005). The Montreal Cognitive Assessment, MoCA: A brief screening tool for mild cognitive impairment. J. Am. Geriatr. Soc..

[33] Dickerson B.C., Atri A., Clevenger C., Karlawish J., Knopman D., Lin P.J., Norman M., Onyike C., Sano M., Scanland S. (2025). The Alzheimer’s Association clinical practice guideline for the Diagnostic Evaluation, Testing, Counseling, and Disclosure of Suspected Alzheimer’s Disease and Related Disorders (DETeCD-ADRD): Executive summary of recommendations for specialty care. Alzheimer’s Dement..

[34] Dickerson B.C., McGinnis S.M., Xia C., Price B.H., Atri A., Murray M.E., Mendez M.F., Wolk D.A. (2017). Approach to atypical Alzheimer’s disease and case studies of the major subtypes. CNS Spectr..

[35] Tang-Wai D.F., Graff-Radford N.R., Boeve B.F., Dickson D.W., Parisi J.E., Crook R., Caselli R.J., Knopman D.S., Petersen R.C. (2004). Clinical, genetic, and neuropathologic characteristics of posterior cortical atrophy. Neurology.

[36] Rabinovici G.D., Furst A.J., Alkalay A., Racine C.A., O’Neil J.P., Janabi M., Baker S.L., Agarwal N., Bonasera S.J., Mormino E.C. (2010). Increased metabolic vulnerability in early-onset Alzheimer’s disease is not related to amyloid burden. Brain.

[37] Shaw L.M., Vanderstichele H., Knapik-Czajka M., Clark C.M., Aisen P.S., Petersen R.C., Blennow K., Soares H., Simon A., Lewczuk P. (2009). Cerebrospinal fluid biomarker signature in Alzheimer’s disease neuroimaging initiative subjects. Ann. Neurol..

[38] Sapolsky D., Bakkour A., Negreira A., Nalipinski P., Weintraub S., Mesulam M.M., Caplan D., Dickerson B.C. (2010). Cortical neuroanatomic correlates of symptom severity in primary progressive aphasia. Neurology.

[39] Sapolsky D., Domoto-Reilly K., Dickerson B.C. (2014). Use of the Progressive Aphasia Severity Scale (PASS) in monitoring speech and language status in PPA. Aphasiology.

[40] Morris J.C. (1993). The Clinical Dementia Rating (CDR): Current version and scoring rules. Neurology.

[41] Kertesz A. (1982). Western Aphasia Battery.

[42] Shewan C.M., Kertesz A. (1980). Reliability and validity characteristics of the Western Aphasia Battery (WAB). J. Speech Hear. Disord..

[43] Rao L.A., Roberts A.C., Schafer R., Rademaker A., Blaze E., Esparza M., Salley E., Coventry C., Weintraub S., Mesulam M.M. (2022). The Reliability of Telepractice Administration of the Western Aphasia Battery-Revised in Persons With Primary Progressive Aphasia. Am. J. Speech Lang. Pathol..

[44] Goodglass H., Kaplan E. (1983). Boston Diagnostic Aphasia Examination Booklet.

[45] Putcha D., Brickhouse M., Touroutoglou A., Collins J.A., Quimby M., Wong B., Eldaief M., Schultz A., El Fakhri G., Johnson K. (2019). Visual cognition in non-amnestic Alzheimer’s disease: Relations to tau, amyloid, and cortical atrophy. Neuroimage Clin..

[46] Primativo S., Yong K.X., Shakespeare T.J., Crutch S.J. (2017). The oral spelling profile of posterior cortical atrophy and the nature of the graphemic representation. Neuropsychologia.

[47] Wang L., St-Georges M.A., Lavoie M., Migliaccio R., Montembeault M. (2024). Language Profile of Posterior Cortical Atrophy: A comparative Study with Alzheimer’s Disease Variants. medRxiv.

[48] Rapcsak S.Z., Henry M.L., Teague S.L., Carnahan S.D., Beeson P.M. (2007). Do dual-route models accurately predict reading and spelling performance in individuals with acquired alexia and agraphia?. Neuropsychologia.

[49] Yong K.X., Warren J.D., Warrington E.K., Crutch S.J. (2013). Intact reading in patients with profound early visual dysfunction. Cortex.

[50] Purcell J., Rapp B., Martin R.C. (2021). Distinct Neural Substrates Support Phonological and Orthographic Working Memory: Implications for Theories of Working Memory. Front. Neurol..

[51] Duricy E., Durisko C., Dickey M.W., Fiez J.A. (2023). Comparing the Reliability of Virtual and In-Person Post-Stroke Neuropsychological Assessment with Language Tasks. Arch. Clin. Neuropsychol..

[52] Groot C., Yeo B.T.T., Vogel J.W., Zhang X., Sun N., Mormino E.C., Pijnenburg Y.A.L., Miller B.L., Rosen H.J., La Joie R. (2020). Latent atrophy factors related to phenotypical variants of posterior cortical atrophy. Neurology.

