# Supplementary material for: The Heterogeneity of Reading and Spelling Deficits in Posterior Cortical Atrophy
Source: Brain Sci. 2025 Oct 28;15(11):1154. doi: 10.3390/brainsci15111154 (PMC12650043; doi:10.3390/brainsci15111154)
Supplement: Supplementary file 1 [file brainsci-15-01154-s001.zip › brainsci-3859702-supplementary.pdf]

Supplementary Materials

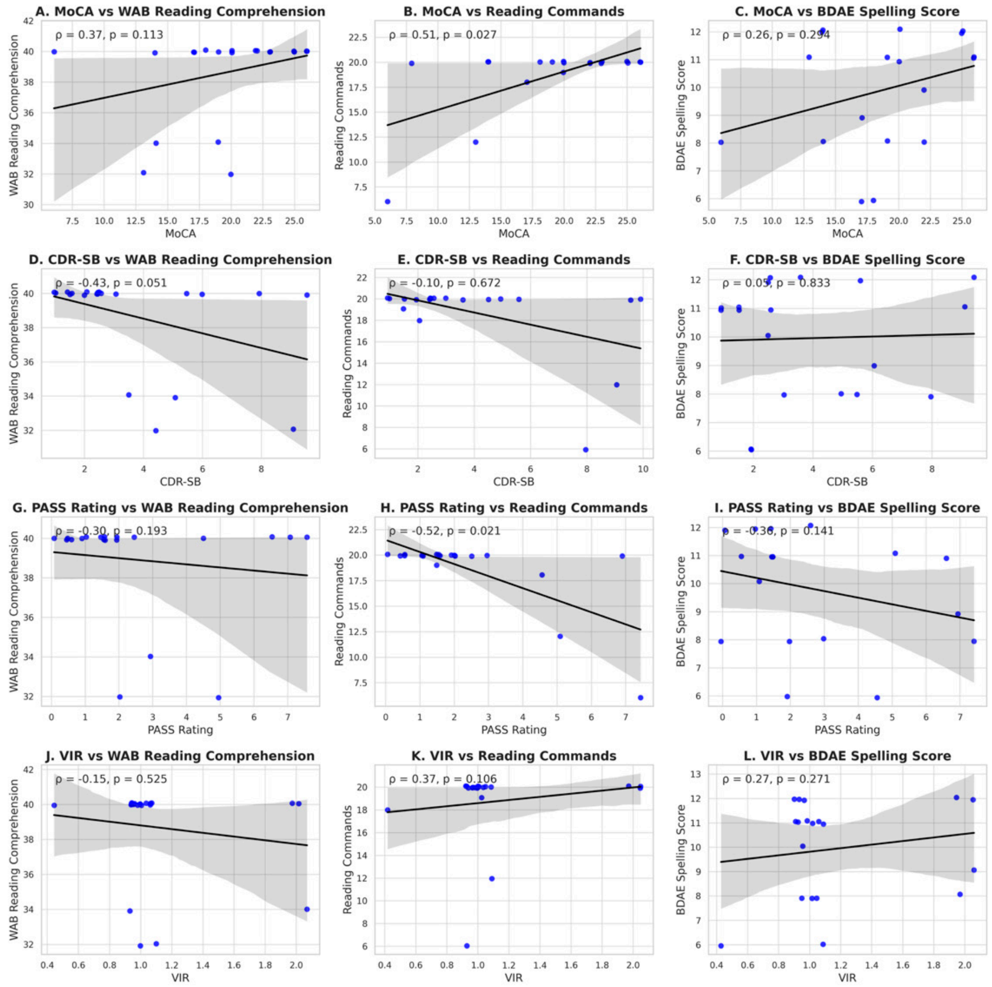

Supplementary Materials Figure S1. Associations between cognitive and clinical severity with tests of reading and spelling. Spearman's correlation coefficient (ρ) and significance (p-value) are shown in each scatterplot depicting the relationships between (**A**) global cognition, measured by the Montreal Cognitive Assessment (**MoCA**) and (A)WAB Reading Comprehension, (**B**) Reading Commands, and (**C**) BDAE Spelling. Also depicted are the associations between global functional impairment measured by the CDR sum-of-box scores (**CDR-SB**) and (**D**) WAB Reading Comprehension, (**E**) Reading Commands, and (**F**) BDAE Spelling. Further, global language impairment measured by the Progressive Aphasia Severity Scale (**PASS**) scores is shown in association with (**G**) WAB Reading Comprehension, (**H**) Reading Commands, and (**I**) BDAE Spelling scores. Last, visuospatial functional impairment scores measured by the Visuospatial Impairment Rating (**VIR**) scores are shown in association with (**J**) WAB Reading Comprehension, (**K**) Reading Commands, and (**L**) BDAE Spelling scores.

Participant	CDR Global	CDR-SB	MoCA	VIR	PASS Sum of Boxes	WAB Reading Comprehension Score	WAB/Informal Reading Commands Score	BDAE Spelling Score
**Participant 1**	0.5	2.5	22/30	1	1	40/40	20/20	10/12
**Participant 2**	1	5	19/30	1	3	34/40	20/20	8/12
**Participant 3**	1	4.5	20/30	1	2	32/40	20/20	N/A*
**Participant 4**	0.5	1.5	20/30	1	1.5	40/40	19/20	11/12
**Participant 5**	1	6	17/30	2	7	40/40 -	N/A**	9/12 ---
**Participant 6**	1	8	6/30	1	7.5	40/40	6/20	8/12
**Participant 7**	1	9.5	14/30	2	0	40/40	20/20	12/12
**Participant 8**	0.5	1	24/30	1	0.5	40/40	20/20	11/12
**Participant 9**	0.5	2	18/30	1	2	40/40	20/20	6/12
**Participant 10**	0.5	2.5	26/30	1	1.5	40/40	20/20	11/12
**Participant 11**	0.5	2.5	25/30	1	1.5	40/40	20/20	12/12
**Participant 12**	1	3.5	14/30	2	1.5	34/40	20/20	12/12
**Participant 13**	0.5	1.5	23/30	1	0.5	40/40	20/20	N/A*
**Participant 14**	2	10	8/30	2	7	4/4 --	16/20	N/A**
**Participant 15**	0.5	3	22/30	1	2	40/40	20/20	8/12
**Participant 16**	1	5.5	20/30	1	1	40/40	20/20	12/12
**Participant 17**	0.5	1	26/30	1	1.5	40/40	20/20	11/12
**Participant 18**	0.5	1.5	19/30	1	6.5	40/40	N/A**	11/12
**Participant 19**	2	9	13/30	1	5	32/40	12/20	11/12
**Participant 20**	0.5	2	17/30	0.5	4.5	40/40	18/20	6/12 ----
**Participant 21**	1	5.5	14/30	2	0	12/12 --	6/17 --	8/12
**Participant 22**	0.5	2.5	25/30	1	2.5	40/40	20/20	12/12
**Participant 23**	0.5	2.5	23/30	1	0.5	40/40	20/20	N/A*

Supplementary Materials Table S1. Individual-level performance scores on clinical measures and tests of reading and spelling.

Non-Standardized Assessment:
*did not complete the BDAE Spelling (was not administered)
**did not complete WAB/Informal Reading Commands (was not administered)
- Clinician read items aloud to participant
-- Task discontinued due to difficulty
--- BDAE Spelling: Spelled words orally
----Switched from written to oral spelling during task
